# Immunohistochemical analysis of expression and allelotype of mismatch repair genes (*hMLH1* and *hMSH2*) in bladder cancer

**DOI:** 10.1054/bjoc.2000.1595

**Published:** 2001-02

**Authors:** H Sh Kassem, J M Varley, S M Hamam, G P Margison

**Affiliations:** CRC Carcinogenesis Group; CRC Cancer Genetics Groups, Paterson Institute for Cancer Research, Christie Hospital, Manchester, M20 4BX, United Kingdom; Pathology Department, Faculty of Medicine Alexandria University, Egypt

**Keywords:** MLH1, MSH2, allelotype, immunohistochemistry, bladder cancer

## Abstract

Mutation of human homologues of DNA mismatch repair (MMR) genes in tumours has been shown to be associated with the phenomenon of microsatellite instability (MSI). Several studies have reported the occurrence of MSI in bladder cancer, but evidence of involvement of MMR genes in the pathogenesis of this cancer is still unclear. We therefore utilized quantitative immunohistochemical (IHC) image analysis and PCR-based allelotype analysis to determine *hMLH1* and *hMSH2* genes alteration in a cohort of Egyptian bladder cancer samples. IHC analysis of 24 TCC and 12 SCC revealed marked- intra and intertumour heterogeneity in the levels of expression of the two MMR proteins. One TCC lost MLH1 expression and one lost MSH2, (1/24, 4%), and one SCC lost MSH2 (1/12, 8%). A large proportion of analysed tumours revealed a percentage positivity of less than 50% for MLH1 and MSH2 expression (44% and 69%, respectively). Complete loss of heterozygosity in three dinucleotide repeats lying within, or in close proximity to, *hMLH1* and *hMSH2* was rare (2/57, (4%) for *MLH1*; and 1/55, (2%) for *MSH2*), however allelic imbalance was detected in 11/57 (*hMLH1*) and 10/55 (*hMSH2*) at any of the informative microsatellite loci. These alterations in structure and expression of DNA MMR genes suggest their possible involvement in the tumorigenesis and/or progression of bladder cancer. © 2001 Cancer Research Campaign http://www.bjcancer.com


*British Journal of Cancer* (2001) **84**(3), 321–328
© 2001 Cancer Research Campaign
doi: 10.1054/ bjoc.2000.1595, available online at http://www.idealibrary.com on IDEAL®

http://www.bjcancer.com

### Immunohistochemical analysis of expression and allelotype of mismatch repair genes (*hMLH1* and *hMSH2*) in bladder cancer

**HSh Kassem[1,3], JM Varley[2], SM Hamam[3] and GP Margison[1]**

[1]CRC Carcinogenesis Group,[2]CRC Cancer Genetics Groups, Paterson Institute for Cancer Research, Christie Hospital, Manchester M20 4BX, United Kingdom, [3]Pathology Department, Faculty of Medicine Alexandria University, Egypt

**Summary** Mutation of human homologues of DNA mismatch repair (MMR) genes in tumours has been shown to be associated with the phenomenon of microsatellite instability (MSI). Several studies have reported the occurrence of MSI in bladder cancer, but evidence of involvement of MMR genes in the pathogenesis of this cancer is still unclear. We therefore utilized quantitative immunohistochemical (IHC) image analysis and PCR-based allelotype analysis to determine *hMLH1* and *hMSH2* genes alteration in a cohort of Egyptian bladder cancer samples. IHC analysis of 24 TCC and 12 SCC revealed marked- intra and intertumour heterogeneity in the levels of expression of the two MMR proteins. One TCC lost MLH1 expression and one lost MSH2, (1/24, 4%), and one SCC lost MSH2 (1/12, 8%). A large proportion of analysed tumours revealed a percentage positivity of less than 50% for MLH1 and MSH2 expression (44% and 69%, respectively). Complete loss of heterozygosity in three dinucleotide repeats lying within, or in close proximity to, *hMLH1* and *hMSH2* was rare (2/57, (4%) for *MLH1*; and 1/55, (2%) for *MSH2*), however allelic imbalance was detected in 11/57 (*hMLH1*) and 10/55 (*hMSH2*) at any of the informative microsatellite loci. These alterations in structure and expression of DNA MMR genes suggest their possible involvement in the tumorigenesis and/or progression of bladder cancer. © 2001 Cancer Research Campaign http://www.bjcancer.com

**Keywords**: MLH1; MSH2; allelotype; immunohistochemistry; bladder cancer

The incidence of bladder cancer varies throughout the world (Boring et al, 1994). The highest incidence rates are generally found in industrially developed countries, particularly North America and Western Europe, and in areas associated with endemic Schistosomiasis infection, including parts of Africa and the Middle East (Parkin et al, 1993). The vast majority (90%) of tumours of the urinary bladder is of urothelial origin, arising from the specialized transitional type of epithelium that lines the bladder. Because of the multipotential nature of the urothelium, tumours of other types; squamous, glandular, and neuroendocrine; can also arise (Brodsky, 1992).

It is generally accepted that, as with most other carcinomas, bladder cancer development is dependent on a combination of genetic and environmental factors. Although no genetic progression model has been defined for bladder cancer, multiple genetic alterations have been identified in primary bladder tumours even at their very early stages (Mao, 1996). Genetic alteration in one or more of three categories of genes has been almost universally connected to cancer development. These include proto-oncogenes, tumour suppressor genes, and genes that function in damage recognition and repair (Lairmore and Norton, 1997).

The existence of mismatch repair (MMR) enzymes in bacteria has been known for at least two decades and more recently similar activities have been identified in yeast and higher eukaryotes (Modrich, 1991). Mutations of MMR genes have been shown to be associated with colorectal and other cancers in the hereditary nonpolyposis colorectal syndrome (HNPCC), as well as a proportion of sporadic colorectal cancers (Lairmore and Norton, 1997). Mutational inactivation of both copies of DNA MMR genes results in a profound repair defect which is presumed to lead to progressive accumulation of secondary mutations throughout the genome, some of which affect important growth regulatory genes and hence give rise to cancer (Peltomaki and de la Chapelle, 1997).

The protein products of MMR genes (MSH2, MLH1, PMS1, PMS2, GTBP or MSH6) act in concert as part of complexes to enable the recognition and excision of mis- or unmatched bases. MSH2 and MLH1 proteins are vital, which may explain why the majority of HNPCC mutations occur in either *MSH2* or *MLH1* (Frayling, 1999). MMR plays an important role in maintaining replication fidelity, processing and regulating recombination (Kolodner, 1995). Mutation in any of the genes required to maintain the integrity of the genome will result in a state of genetic instability, a 'mutator' phenotype, which could contribute to the accumulation of mutations required for multistage carcinogenesis (Loeb, 1991, 1994).

Microsatellites are stably inherited short tandem repeat DNA sequences that are unique to each individual (Brentnall, 1995). Microsatellite instability (MSI) appears as aberrant-sized alleles resulting from a gain or loss of short repeat units in tumour DNA in comparison to normal DNA. MSI serves as a useful marker of a mutator phenotype (Peltomaki, 1997) which is a characteristic of HNPCC (Aaltonen et al, 1993, 1994) and reflects malfunctions in the replication or repair of DNA (Aaltonen et al, 1993). Biochemical studies have provided a link between MSI and defective MMR (Parsons et al, 1993). In addition to HNPCC, MSI has been observed in several sporadic tumours (Han et al, 1993;

*Received 12 November 1999*
*Revised 8 March 2000*
*Accepted 8 May 2000*

*Corespondence to:* GP Margison

Chong et al, 1994; Wada et al, 1994; Speicher, 1995). Several studies have reported the occurrence of MSI in bladder cancer (Gonzalez-Zulueta et al, 1993; Mao et al, 1994, 1996; Orlow et al, 1994; Uchida et al, 1996; Steiner et al, 1997; Christensen et al, 1998; Mourah et al, 1998). Recently, the tumour spectrum associated with germline mutations of MMR genes in HNPCC cases was found by Aarnio et al (1999) to involve several organs including the uro-epithelium. These studies may point to the possibility of involvement of mismatch repair genes in sporadic bladder cancer, thus the authors were interested in assessing MMR status in bladder cancer in the context of quantitative expression and detection of microsatellite instability at the regions of the MMR genes: *MSH2* and *MLH1*.

### MATERIALS AND METHODS

### Samples

Fifty seven bladder tumour samples (44 TCC and 13 SCC) were obtained from radical cystectomy specimens from the Department of Urology, Faculty of Medicine, Alexandria University. The patients' mean age was 56.7 ± 8.6 years (ranging from: 38–71 years). Forty three patients were male (75%) and 14 were female (25%). Sixty eight percent of patients (39/57) self-reported a history of either a positive test (presence of bilharzial eggs in urine) or past treatment for schistosomiasis. Tissues collected included grossly obvious tumour tissue that was confirmed by subsequent histopathological diagnosis at the Pathology Department, Faculty of Medicine, Alexandria University. Samples were immediately frozen on dry ice, stored at –70˚C and were transferred on dry ice to the Paterson Institute for Cancer Research in Manchester where they were analysed. DNA was extracted from paired frozen bladder samples (grossly obvious tumour and macroscopically non-involved mucosa obtained as far from the site of tumour as possible) using proteinase K digestion and standard phenol chloroform extraction method (Sambrook et al, 1989). After taking the tissue for DNA extraction, those samples that were large enough were fixed in 4% buffered formaldehyde and processed to paraffin wax. Representative haematoxylin and eosin stained sections were examined microscopically to confirm the presence of tumour, and to assess the percentage of tumour cells. All cases selected showed at least 40% neoplastic cells amongst fibrous and muscular tissues.

### Immunohistochemical analysis of MLH1 and MSH2 proteins

36 analysable samples (24 TCC and 12 SCC) were selected from the formalin-fixed, paraffin embedded sections and serial 3 μm thick sections were cut and subbed on APES (3-aminopropyltriethoxysilane from Sigma) coated slides. Sections were deparaffinized through xylene and graded alcohol. The sections were then treated by microwaving to retrieve antigens in 1 litre of 10 mM citrate buffer (pH 6.0) for 30 min at 700 W, and allowed to cool. Endogenous peroxidase activity was blocked by incubating in 1% $H_2O_2$/100% methanol for 30 min. Non-specific protein binding was eliminated by incubation for 30 min at room temperature in 5% normal rabbit serum (Dako). Sections were incubated overnight at 4˚C with the primary antibody at a dilution of 5 μg $ml^{-1}$ and 10 μg $ml^{-1}$ of mouse monoclonal antihuman MLH1 and MSH2 immunoglobulins, respectively (PharMingen). Sections were then incubated with biotinylated rabbit anti-mouse antibody (Dako). Binding sites were visualized using standard avidin–biotin complex method (Hsu et al, 1981). Sections were counterstained with propidium iodide 2.5 μg $ml^{-1}$ (Sigma). Positive controls consisting of sections of human colon and negative controls, in which TBS was used instead of primary antibody, were included with every batch. Initially, samples were subjectively screened and this was followed by analysis of 5–10 fields (depending on the section size) using Lucia G image analysis software package which automates data collection. The negative control section for each sample was used to set a threshold above which staining in the immune-stained sections was defined by the analysis system as positive. Thus any background staining was excluded. The total number of tumour cells was determined in the analysed fields by applying a series of semi-automated steps which counts positive-staining cells then adds this number to the negative-staining tumour cells which are counted by virtue of their propidium iodide fluorescence due to lack of the quenching effect of the DAB in the unstained cells. The image analysis thus provides a measure of the total and the positive-stained cells from which the percentage positivity is calculated.

### Microsatellite analysis at *hMSH2* and *hMLH1* loci

A PCR-based method was used to assess allellic loss and/or alterations occurring at loci which lie within, or in close proximity to, *MSH2* (2p16) and *MLH1* (3p21) genes. Three dinucleotide microsatellite repeats on chromosome 2p16 (D2S123, D2S391, D2S119) and 3p21 (D3S1612, D3S1611, D3S1561) were used. Primer sequences were taken from the 1993–94 Genethon human genetic linkage map (Gyapay et al, 1994).

PCR amplification was carried out in a 10 μl reaction mix using standard microsatellite analysis PCR method (Varley et al, 1999). Annealing temperature for D3S1611 marker was adjusted at 62˚C rather than 56˚C to eliminate non-specific bands. Products were separated on 6% denaturing polyacrylamide sequencing gels (National Diagnostics) and dried before exposure overnight to Fuji RX film at -70˚C. Autoradiographs were scored by 2 independent examiners. Loss of heterozygosity (LOH) was scored when there was complete loss of one allele in an informative sample (i.e. the normal DNA was heterozygous), and allelic imbalance (AI) was scored when there was an observed difference in intensity of the alleles between tumoral and normal DNA. Change in allele size or the appearance of a new band in tumoral DNA was scored as new allele (NA). Only informative samples at any of the loci analysed for each gene were included in the calculation of the frequency of allelic loss.

### RESULTS

### Immunohistochemical analysis of MSH2 and MLH1 proteins

Immunohistochemical staining of MSH2 and MLH1 showed exclusively nuclear staining in both normal and tumour cells. IHC staining of samples of normal urothelium from non-invovled mucosa obtained from radical cystectomy specimens showed relative homogeneity of expression of MMR proteins (Figure 1) with basal, middle and superficial cells all showing positive staining. Metaplastic squamous epithelium of the bladder showed staining of basal cells, most of the middle cell layer (prickle zone) but with faint or even absent staining of the most superficial flattened cell

 © 2001 Cancer Research Campaign

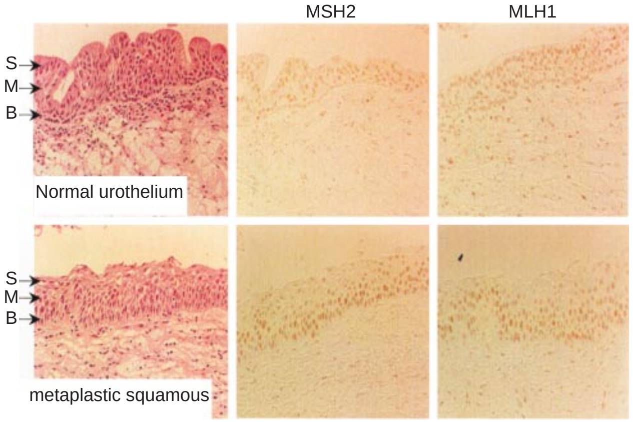

**Figure 1** Immunohistochemical staining of normal urothelium (top row) and metaplastic squamous epithelium (bottom row) for MSH2 and MLH1, (×20 magnification). The superficial (S), middle (M), and basal (B) cell layers are indicated by arrows Colour version available online at http://www.idealibrary.com and http://www.paterson.man.ac.uk/carcinogenesis

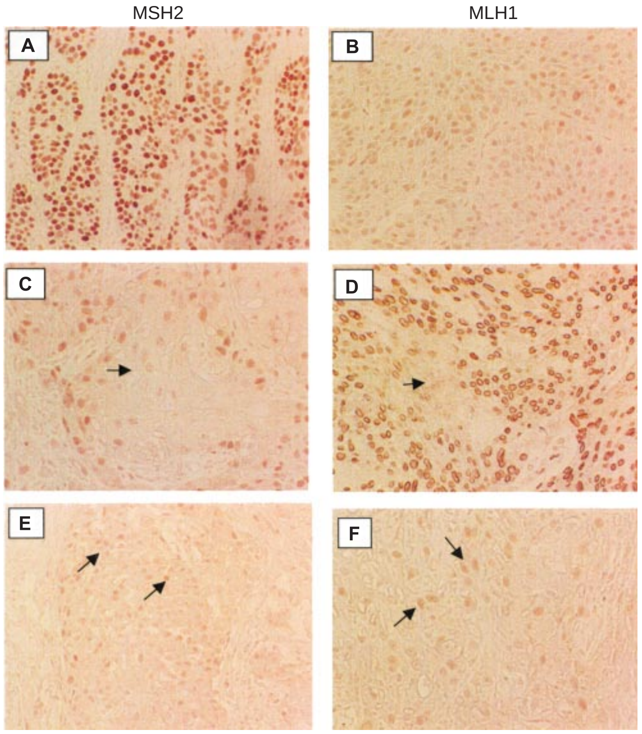

**Figure 2** Representative MSH2 and MLH1 immunostained tumour sections (**A**, **B**, **D**, **E** are TCCs and C and F are SCCs), (x20 magnification). Note the heterogeneity in the level of expression between different tumour sections. Regional (**C** and **D**) and intercellular (**E** and **F**) heterogeneity observed within the same tumour marked by arrows in (**C**, **D**, **E**, and **F**) and explained in detail in the text. Colour version available online at http://www.idealibrary.com and http://www.paterson.man.ac.uk/carcinogenesis layer, distinctly contrasting with the same zone in the normal urothelium.

Initial subjective screening of tumour sections revealed inter- and intratumoral heterogeneity (Figure 2). The staining pattern varied considerably amongst different tumour samples. Some tumours stained relatively homogeneously as shown in Figure 2 (A and B). Heterogeneity within the tumours was frequently observed for both MSH2 and MLH1 in which clusters of cells showed low or relatively absent staining amongst positive-stained cells as shown in Figure 2 (C and D), or occasional positive cells might be found randomly in a generally low expressing tumour as shown in Figure 2 (E and F). Although complete loss of expression of MSH2 and MLH1 was infrequently encountered (one SCC (1/12, 8%) and one TCC (1/24, 4%) lost MSH2 and one TCC lost MLH1 (1/24, 4%), a large proportion of tumours revealed less than 50% positive-stained nuclei for both MSH2 and MLH1 (69% and 44% respectively).

The number of positively stained cells was determined using a threshold defined by the use of tris-buffered saline in place of the immune serum. A wide range of expression of MMR proteins was observed in both pathologic types of bladder cancer (Figure 3). There was no correlation between expression of MSH2 and MLH1

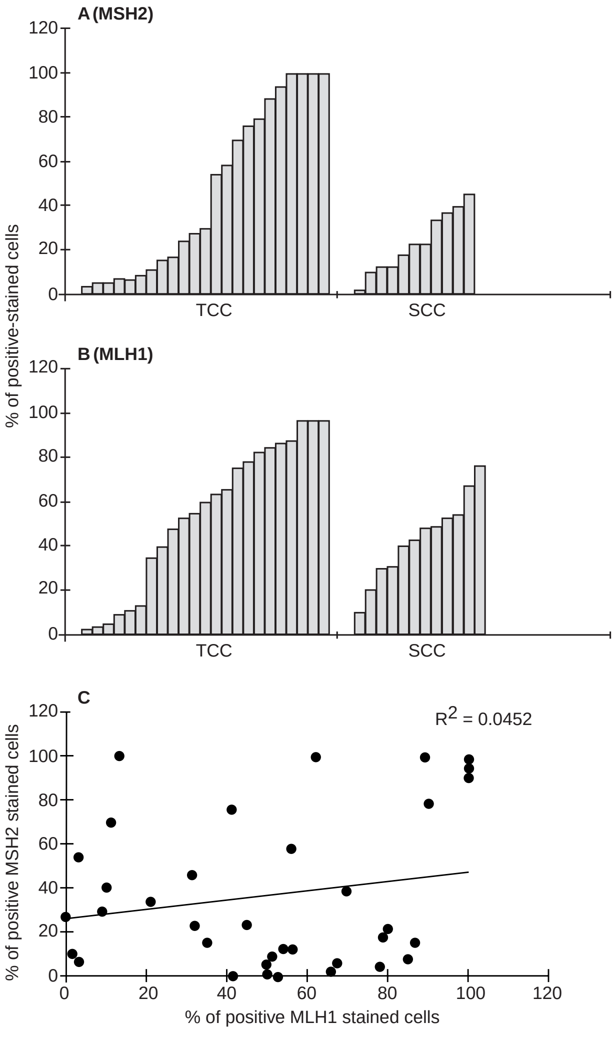

**Figure 3** Intertumoural heterogeneity and wide range of expression of MSH2 (**A**) and MLH1 (**B**) expression in TCC and SCC, and the relationship between both mismatch repair proteins in all samples analysed (**C**)

*© 2001 Cancer Research Campaign*

**Table 1** Percentage positivity of MMR proteins expression in TCC and SCC samples

| MMR protein | Percentage positivity | TCC (24) | SCC (12) | Total (36) |
|---|---|---|---|---|
| MSH2 | Range | 0–100% | 0–46% | 0–100% |
| | Mean | 44.88 | 21.58 | 37.11 |
| | Median | 28.00 | 20.50 | 23.50 |
| | SD | 38.78 | 15 | 34.40 |
| | Coefficient of variation | 86% | 70% | 93% |
| MLH1 | Range | 0–100% | 10–79% | 0–100% |
| | Mean | 53.46 | 45.17 | 50.69 |
| | Median | 59.00 | 47.50 | 52.50 |
| | SD | 35.79 | 19.63 | 31.28 |
| | Coefficient of variation | 67% | 43% | 62% |

(Figure 3). Table 1 summarizes the findings in the two pathologic types. The coefficient of variation for both proteins was high, confirming the observer-reported intertumoral heterogeneity within TCC and SCC samples. The mean percentage positivity of MSH2 in SCC was observed to be half that of MLH1 in the same pathologic subtype; this proved to be statistically significant ($P = 0.02$ using Wilcoxon Signed Ranks test) (Table 1). It was also observed that the mean percentage positivity of MSH2 in SCC was lower than that in TCC, however this was not found to be statistically significant ($P = 0.2$ using the Mann-Whitney 2-tailed test) (Table 1). The relationship between the bilharzial status and the levels of expression of MLH1 and MSH2 was studied using the Mann-Whitney test and revealed a statistically significant difference in the level of expression of MLH1 but not MSH2 in the schistosomal positive versus negative samples ($P = 0.01$, 0.9 respectively). The samples were further stratified into TCC and SCC in relation to the bilharzial status and a significant difference was still observed in SCC but not in the TCC group ($P = 0.04$, 0.15, respectively).

### Results of microsatellite analysis

Allelic loss was assessed at the markers flanking *hMSH2* on chromosome 2p16 and *hMLH1* on 3p21 using microsatellite repeats as close together as possible to detect small regions of deletion (Gyapay et al, 1994; Tomlinson et al, 1996). Representative autoradiographs of the gels used for scoring in this study are shown in Figure 4. Tables 2A and 2B list the allellotypes of samples showing any alterations at the microsatellite loci studied

**Table 2** The allelotypes of those samples showing alterations at (A) 2p16 and (B) 3p21, and the expression of the corresponding MMR protein

**A**

| Sample code | Microsatellite loci at 2p16 | | | IHC of MSH2 (% positivity) |
|---|---|---|---|---|
| | D2S123 | D2S391 | D2S119 | |
| | 3 cM | 5 cM | | |
| H1 | NI | AI | AI | 8 |
| H7 | NI | AI | NI | ND |
| H13 | NI | AI | AI | 27 |
| H30 | ROH | AI | AI | 13 |
| A4 | AI | ROH | ROH | 100 |
| A9 | NI | AI | AI | 54 |
| A11 | ROH | NI | AI | 37 |
| A21 | LOH | NA | NA | ND |
| A29 | ROH | AI | NI | 34 |
| A32 | AI | AI | NI | ND |
| A35 | AI | ROH | NI | 46 |

**B**

| Sample code | Microsatellite loci at 3p21 | | | IHC of MLH1 (% positivity) |
|---|---|---|---|---|
| | D3S1612 | D3S1611 | D3S1561 | |
| | 0 cM | 0 cM | | |
| H1 | NI | NI | AI | 85 |
| H7 | AI | ROH | NI | ND |
| H22 | NI | ROH | AI | 42 |
| H27 | AI | AI | NI | 4 |
| H30 | ROH | AI | NI | 55 |
| A4 | LOH | LOH | LOH | 89 |
| A10 | NI | NI | AI | 87 |
| A11 | AI | AI | NI | 70 |
| A21 | LOH | NA | NA | ND |
| A29 | ROH | AI | NI | 21 |
| A33 | AI | AI | NI | ND |
| A34 | AI | AI | NI | 100 |
| A35 | AI | AI | ROH | 31 |
| A9 | ROH | ROH | NA | 3 |

ROH: retention of heterozygosity, NI: non-informative for heterozygosity, AI: allelic imbalance, LOH: loss of heterozygosity, NA: new allele or shift of size of an existing allele, ND: not done.

 *© 2001 Cancer Research Campaign*

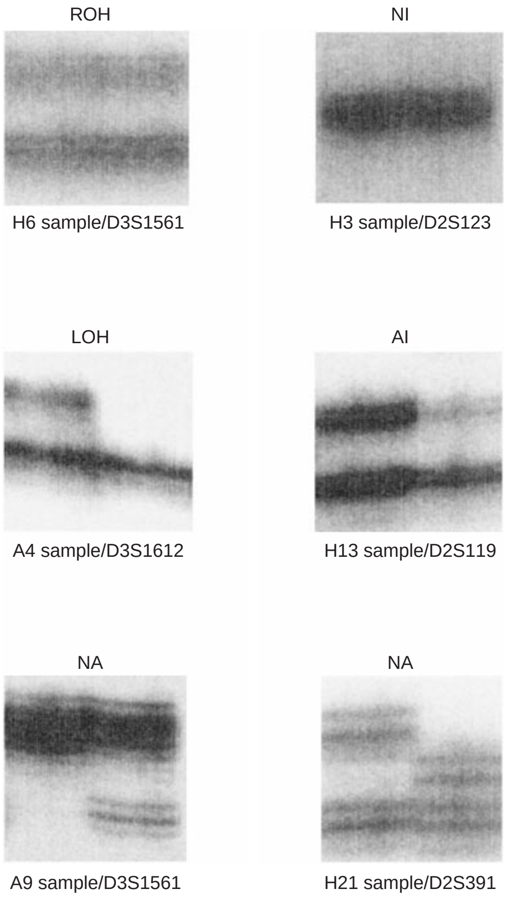

**Figure 4** Representative autoradiographs of the scoring used in this study. ROH: retention of heterozygosity, NI: non-informative of heterozygosity, LOH: loss of heterozygosity, AI: allelic imbalance, NA: new allele (appearance of a new allele or change of size of an existing allele). NB: in each case normal DNA (extracted from non involved mucosa) is in the left lane and tumour DNA is in the right lane along with their corresponding immunohistochemical status expressed as percentage positivity.

Complete LOH was infrequently encountered. Only one TCC sample (A21; 1/42 i.e. 2%) of the informative samples revealed LOH at one of the markers on chromosome 2p (D2S123). Two TCC samples revealed LOH at chromosome 3p markers (2/44 (5%)), one sample (A4) revealed LOH at the 3 loci analysed while the other sample (A21), revealed LOH at D3S1612 only. Complete LOH was not detected in any of the SCC samples analysed. The incidence of AI was, however, higher than LOH. This could be due to heterogeneity within the tumour and allelic loss occurring in a subpopulation of tumour cells and therefore would be in agreement with the observed intratumoral heterogeneity in expression of MMR proteins. An alternative explanation is that DNA was extracted from visible tumour tissue allowing contamination by normal cells. The frequency of allelic loss (AI and LOH) at the markers flanking *MSH2* and *MLH1* genes was higher in SCC (38% for both genes) than in TCC (14% and 18%, respectively), but the difference was not statistically significant using Fisher's Exact test ($P$ = 0.3 and 0.5, respectively). There was no effect of the bilharzial status on the incidence of microsatellite alterations at the studied loci (Fisher's Exact test, $P$ = 1).

Allelic imbalance and LOH occurred at markers compatible with the involvement of *hMSH2* and *hMLH1* genes. AI was

**Table 3** Summary of the correlation of IHC (percentage positivity) and microsatellite alteration at 2p16 and 3p21

| **Allelotype of loci on 2p16 and 3p21** | **Mean percentage positivity**[a] | |
|---|---|---|
| | **MSH2** | **MLH1** |
| No change ($n$ = 21) | 38.5 | 52.5 |
| Changes present on either 2p or 3p markers ($n$ = 12) | 36.7 | 48.9 |
| Changes at 2p ($n$ = 8) | 40 | 44 |
| Changes at 3p ($n$ = 11) | 37.6 | 53.4 |

[a]The means of the percentage positivity for each group.

detected in the furthest marker studied on chromosome 3p21 in sample H22, however, the IHC finding supported the possibility of involvement of *MLH1* in a sub-population of these tumours as the percentage positivity was 42%. Samples that were found to be negative for expression of MSH2 (H12, H22) and MLH1 (H13) did not reveal AI at the flanking microsatellite loci, this could be due to the fact that those samples were non-informative at the studied loci or due to another mechanism of mutation of the involved MMR gene. Sample A4 showed LOH at loci flanking *MLH1*, and whilst IHC showed a high percentage positivity (89%) for this protein, the intensity of staining was very faint. This may suggest a structural alteration in the expressed protein with reduced affinity for the antibody rather than a technical problem as this sample stained very strongly for MSH2. There was no observed difference in the means of the percentage positivity of expression of MMR proteins in relation to the occurrence of alterations at the loci flanking each or both of the genes (Table 3). Thus, the two methods of analysis independently detected possible alterations in structure and/or expression of *MSH2* and *MLH1* genes.

The appearance of a new allele or shift of an existing allele (NA) was detected in two samples. One of them (A21) revealed NA at both 2p16 and 3p21 indicating the presence of a potential mutator phenotype in this tumour which also showed LOH at one of the loci flanking both *hMSH2* and *hMLH1*. To exclude the possibility of PCR contamination or crossing of samples, DNA was again extracted from the original tissues of this sample (tumour and mucosa) and the results were confirmed. Unfortunately, tumour tissue was not available to study the expression of MMR proteins in this sample. The samples analysed in this study were all advanced bladder cancer (at least stage T2 and grades 2 and 3), so it was not possible to assess MMR status in the early stages of bladder cancer from the cohort studied.

### DISCUSSION

Earlier studies have suggested that MMR deficiency results in an increased rate of spontaneous mutation, giving the hallmark of a mutator phenotype (Cox, 1973, 1976). MSI serves as a useful marker of the mutator phenotype that is characteristic of HNPCC (Aaltonen et al, 1993, 1994) and has been attributed to mutations in one of several MMR genes including *hMSH2, hMLH1, hPMS1 and hPMS2* (Leach et al, 1993; Bronner et al, 1994; Fishel et al, 1994; Nicolaides et al, 1994; Papadopoulos et al, 1994). MSI has been described in a variety of sporadic cancers, such as colon (Thibodeau et al, 1993), endometrium (Risinger et al, 1993) pancreas and stomach (Han et al, 1993) and prostate (Uchida et al, 1996). Previous studies have also reported a high frequency of

*© 2001 Cancer Research Campaign* 

MSI in bladder cancer (Mao et al, 1996; Steiner et al, 1997; Mourah et al, 1998). The finding of MSI in a varying proportion of sporadic cancers suggests that the same type of genetic defects in MMR genes that underlie HNPCC can occur somatically in some sporadic cancers including bladder cancer. However, the proportion of sporadic tumours in which MMR genes have been inactivated has not yet been clearly established (Kane et al, 1997). This might be due to the fact that the methods available for detecting mutations in these genes are laborious, and no single method is capable of detecting all the types of mutations that could exist. This is particularly problematic when tumour samples are analysed because of the contamination of tumour cells with normal tissue in such samples and also due to the fact that the complete spectrum of mutations and modes of inactivation applicable to the analysis of these genes is not currently known (Kane et al, 1997). In a clinical setting, the use of IHC appears to offer a relatively convenient and rapid method for prescreening tumours for defects in the expression of mismatch repair genes (Thibodeau et al, 1996).

In the present study alterations in *hMSH2* and *hMLH1* were determined in TCC and SCC of the bladder using quantitative immunohistochemistry and PCR-based microsatellite analysis methods. IHC analysis showed nuclear localization of staining as reported in previous studies (Lim et al, 1996; Thibodeau et al, 1996; Fink et al, 1997). Immunostaining of normal urothelium revealed a relatively homogeneous staining of almost all cells as shown in Figure 1. This is the first report of the pattern of staining of the normal urothelium for these MMR proteins. Intratumoral heterogeneity was observed in the majority of samples. Intratumoral heterogeneity in *MLH1* and *MSH2* gene alterations has previously been reported by Habano et al (1998) in sporadic colorectal cancer using a sensitive technique for allelic loss detection in different crypts within the same tumour after microdissection. This is therefore in agreement with our findings using immunohistochemistry, which has the benefits of being simpler and more convenient. Metaplastic squamous epithelium showed MSH2 and MLH1 staining of the basal cells and most of the prickle cell layer which is comparable to that previously reported for MSH2 staining of the squamous epithelial lining of the oesophagus and MSH2 and MLH1 staining of the oral mucosa (Leach et al, 1996 and Lo Muzio et al, 1999, respectively).

The absence of MLH1 and MSH2 immunostaining was previously correlated with the presence of mutation in the corresponding gene in sporadic colon cancer (Thibodeau et al, 1996). Such complete absence of expression of MSH2 and MLH1 was infrequently encountered in the present study. However, a large proportion of tumours revealed a percentage positivity of less than 50% for MSH2 and MLH1 (69% and 44%, respectively). The statistically significant lower level of expression of MSH2 in comparison to MLH1 in SCC suggests the possible greater involvement of MSH2 than MLH1 in SCC tumorigenesis and/or progression. The statistically significant difference in the expression of MLH1 in relation to the bilharzial status might suggest a possible role of schistosomiasis in altering the expression of MLH1 in SCC, however this should be further investigated.

In HNPCC, defective MMR is a very early event (Parsons et al, 1995), while in sporadic non-small cell lung carcinoma it has been suggested that defective MMR is a later event related to lung tumour progression (Wieland et al, 1996). The present study suggests that MMR deficiency in bladder cancer is likely to occur during tumour progression rather than as an initiating event since the majority of the samples analysed revealed intratumoral heterogeneity in expression of MMR proteins rather than complete loss in all cells of the tumour. The present study involved only advanced stages (at least T2) and advanced grades (2 and 3) of bladder cancer. In this patient group it was therefore not possible to correlate the MMR involvement with tumour progression. The observed intertumoral variation in the expression of these proteins might have prognostic implications regarding outcome or response to therapy. Jin et al (1999) suggested that reduction in expression of MSH2 in TCC of the bladder might be useful in predicting recurrence.

Tumour suppressor and MMR genes share the requirement for homozygosity at the cellular level and somatic deletion is a common event in both (Hemminki et al, 1994). This second mutational step may be revealed as LOH, which was previously reported at the *hMLH1* locus in HNPCC (Hemminki et al, 1994) as well as at *hMLH1* and *hMSH2* loci in sporadic colorectal cancers (Tomlinson et al, 1996; Benachenhou et al, 1998b), in non-small cell lung cancer (Benachenhou et al, 1998a) and in breast cancer (Benachenhou et al, 1999). Christensen et al (1998) reported the occurrence of LOH at microsatellites located close to *hMSH2* and *hMLH1* and suggested a possible role of these genes in causing profound MSI in bladder cancer. Allelic loss at 3p was previously reported by Rosin et al (1995) and Li et al (1996) which may suggest involvement of *hMLH1* in TCC of the bladder. However these studies analysed only TCC and did not include other methods of analysis to confirm *hMLH1* involvement. In the present study, allelic loss was studied at loci (D2S123, D2S391 and D2S119) which are known to be linked to *hMSH2* (Leach et al, 1993). D3S1611 is known to lie within an intron of *hMLH1* (Papadopoulos et al, 1994). Benachenhou et al (1998a) found that the shortest region of overlapping deletions for 3p21 was delimited by D3S1561 and D3S1612. Allelic loss (LOH or AI) was detected in TCC (14% on 2p16 and 18% on 3p21) and in SCC (38% on 2p16 and 38% on 3p21). However, the frequency of allelic loss in the present study should be considered conservative since DNA was not extracted by microdissection and hence some allelic losses could have been masked by contaminating material from normal cells. The frequency of allelic loss at *MLH1* in TCC (18%) was similar to that found in sporadic colorectal cancer (17%; Benachenhou et al, 1998b and 16%; Tomlinson et al, 1996). Whereas in SCC the frequency of allelic loss at *MLH1* was higher (38%), which was comparable with that detected in breast cancer (46%; Benachenhou et al, 1999) and non-small cell lung cancer (43%; Wieland et al, 1996). The frequency of *MSH2* allelic loss in TCC detected in this study (14%) was comparable to that detected in non-small cell cancer (11.5%; Benachenhou et al, 1998a) and also in sporadic colorectal cancer (15%; Benachenhou et al, 1998b). A higher frequency of allelic loss at *MSH2* was detected in SCC of the bladder (38%). This together with the finding of a low mean percentage positivity of expression of MSH2 in this pathologic type points to the possibility of a contributing role of *MSH2* gene in the pathogenesis of SCC of the bladder.

Other alterations such as small deletions, point mutations, gene rearrangements or DNA cytosine methylation, if they also cause inactivation of both copies of these genes, would escape detection by this PCR based allelic loss analysis method. Therefore, IHC analysis was used to detect samples with deficiency in MMR that was not detected by allelic loss analysis. H12 and H22 lacked expression of MSH2 and H13 lacked MLH1 expression, even though they had no evidence of LOH/AI of the corresponding gene.

 *© 2001 Cancer Research Campaign*

This may be explained by the fact that MMR genes have no mutational hot spots and lack of expression can occur without detectable mutations (Liu et al, 1994). DNA methylation has been reported to be a mode of *hMLH1* gene inactivation in sporadic colon and gastric carcinoma (Kane et al, 1997 and Leung et al, 1999, respectively). A similar mechanism of inactivation by promotor methylation, or possibly by another mechanism of mutation might be responsible for the lack of expression of MMR proteins in the present samples. The samples scored as AI did display reduced expression of the corresponding MMR gene in the majority of cases, however there were exceptions. Sample A34 showed AI at microsatellite loci flanking *hMLH1*, yet expressed high levels of the protein, this however does not exclude the possibility of functional disturbance of the expressed protein and another explanation may be that the structural alteration may have not involved the antibody recognition sites. Also, although sample A4 revealed LOH at all loci on chromosome 3p and whilst the percentage positivity for expression of MLH1 was 89%, the intensity of staining was very low, suggesting abnormal expression. As shown in Table 3 there was no relationship between the expression (percentage positivity) of the MMR proteins and the occurrence of microsatellite alterations at the corresponding chromosome location. This suggests that the two methods of analysis were useful in determining changes that would not have been detected by either of them alone.

In conclusion, this study reveals the occurrence of alterations in the structure and expression of DNA mismatch repair genes and suggests the possibility of involvement of these genes in tumorigenesis and/or progression of TCC and SCC of the bladder. IHC offers a convenient and a rapid method for prescreening tumours for defects in mismatch repair genes, but might not exclude the structural and functional alterations of these proteins. Further extensive studies would be required to establish whether or not in bladder cancer and in other tumour types IHC may indeed be a more reliable diagnostic test for MMR involvement. To the best of our knowledge, this is the first study to demonstrate an involvement of *hMLH1* in TCC and SCC and of *hMSH2* in SCC of the bladder.

### ACKNOWLEDGEMENTS

This work was funded by a scholarship from the Egyptian Government and the Cancer Research Campaign.

### REFERENCES

Aaltonen LA, Peltomaki P, Leach FS, Sistonen P, Pylkkanen L, Mecklin JP, Jarvinen H, Powell SM, Jen J,and Hamilton SR et al (1993) Clues to the pathogenesis of familial colorectal cancer. *Science* **260**: 812–816

Aaltonen LA, Peltomaki P, Mecklin JP, Jarvinen H, Jass JR, Green JS, Lynch HT, Watson P, Tallqvist G, and Juhola M et al (1994) Replication errors in benign and malignant tumors from hereditary nonpolyposis colorectal cancer patients. *Cancer Res* **54**: 1645–1648

Aamio M, Sankila R, Pukkala E, Salovaara R, Aaltonen LA, de la Chapelle A, Peltomaki P, Mecklin JP and Jarvinen HJ (1999) Cancer risk in mutation carriers of DNA-mismatch-repair genes. *Int J Cancer* **81**: 214–218

Benachenhou N, Gulral S, Gorska Flipot I, Labuda D and Sinnett D (1998a) High resolution deletion mapping reveals frequent allelic losses at the DNA mismatch repair loci hMLH1 and hMSH3 in non-small cell lung cancer. *Int J Cancer* **77**: 173–180.

Benachenhou N, Guiral S, Gorska Flipot I, Michalski R, Labuda D and Sinnett D (1998b) Allelic losses and DNA methylation at DNA mismatch repair loci in sporadic colorectal cancer. *Carcinogenesis* **19**: 1925–1929

Benachenhou N, Guiral S, Gorska Flipot I, Labuda D and Sinnett D (1999) Frequent loss of heterozygosity at the DNA mismatch-repair loci hMLH1 and hMSH3 in sporadic breast cancer. *Br J Cancer* **79**: 1012–1017

Boring CC, Squires TS, Tong T and Montgomery S (1994) Cancer statistics, 1994. *CA Cancer J Clin* **44**: 7–26.

Brentnall TA (1995). Microsatellite instability. Shifting concepts in tumorigenesis. *Am J Pathol* **147**: 561–563

Brodsky GL (1992) Pathology of bladder carcinoma. *Hematol Oncol Clin North AM* **6**: 59–80.

Bronner CE, Baker SM, Morrison PT, Warren G, Smith LG, Lescoe MK, Kane M, Earabino C, Lipford J, Lindblom A, Tennergard P, Bollag R, Godwin A, Ward D, Nordenskjold M, Fishel R, Kolodner R and Liskay R (1994) Mutation in the DNA mismatch repair gene homologue hMLH1 is associated with hereditary non-polyposis colon cancer. *Nature* **368**: 258–261.

Chong JM, Fukayama M, Hayashi Y, Takizawa T, Koike M, Konishi M, Kikuchi Yanoshita R and Miyaki M (1994) Microsatellite instability in the progression of gastric carcinoma. *Cancer Res* **54**: 4595–4597

Christensen M, Jensen MA, Wolf H and Omtoft TF (1998) Pronounced microsatellite instability in transitional cell carcinomas from young patients with bladder cancer. *Int J Cancer* **79**: 396–401

Cox EC (1973) Mutator gene studies in *Escherichia coli*: the mut T gene. *Genetics* **73**: 67–80.

Cox EC (1976) Bacterial mutator genes and the control of spontaneous mutation. *Annu Rev Genet* **10**: 135–156

Fink D, Nebel S, Aebi S, Zheng H, Kim HK, Christen RD and Howell SB (1997) Expression of the DNA mismatch repair proteins hMLH1 and hPMS2 in normal human tissues. *Br J Cancer* **76**: 890–893

Fishel R, Lescoe MK, Rao MR, Copeland NG, Jenkins NA, Garber J, Kane M and Kolodner R (1994) The human mutator gene homolog MSH2 and its association with hereditary nonpolyposis colon cancer. *Cell* **77**: 167

Frayling I (1999) Microsatellite Instability *Gut* **45**: 1–4.

Gonzalez-Zulueta M, Ruppert JM, Tokino K, Tsai YC, Spruck III CH, Miyao N, Nichols PW, Hermann, GG, Horn T and Steven K et al (1993) Microsatellite instability in bladder cancer. *Cancer Res* **53**: 5620–5623

Gyapay G, Morissette J, Vignal A, Dib C, Fizames C, Millasseau P, Marc S, Bernardi G, Lathrop, M and Weissenbach J (1994) The 1993–94 Genethon human genetic linkage map. *Nat Genet* **7**: 246–339

Habano W, Sugai T and Nakamura S (1998) Mismatch repair deficiency leads to a unique mode of colorectal tumorigenesis characterized by intratumoral heterogeneity. *Oncogene* **16**: 1259–1265

Han HJ, Yanagisawa A, Kato Y, Park JG and Nakamura Y (1993) Genetic instability in pancreatic cancer and poorly differentiated type of gastric cancer. *Cancer Res* **53**: 5087–5089

Hemminki A, Peltomaki P, Mecklin JP, Jarvinen H, Salovaara R, Nystrom Lahti M, de la Chapelle A and Aaltonen LA (1994) Loss of the wild type MLH1 gene is a feature of hereditary nonpolyposis colorectal cancer. *Nat Genet* **8**: 405–410

Hsu, SM, Raine L, Fanger H (1981) Use of avidin-biotin-peroxidase complex (ABC) in immunoperoxidase techniques: a comparison between ABC and unlabeled antibody (PAP) procedures. *J Histochem Cytochem* **29**: 577–80

Jin TX, Furihata M, Yamasaki I, Kamada M, Liang SB, Ohtsuki Y and Shuin T (1999) Human mismatch repair gene (hMSH2) product expression in relation to recurrence of transitional cell carcinoma of the urinary bladder. *Cancer* **85**: 478–484

Kane MF, Loda M, Gaida GM, Lipman J, Mishra R, Goldman H, Jessup JM and Kolodner R (1997) Methylation of the hMLH1 promoter correlates with lack of expression of hMLH1 in sporadic colon tumors and mismatch repair-defective human tumor cell lines. *Cancer Res* **57**: 808–811

Kolodner RD (1995) Mismatch repair: mechanisms and relationship to cancer susceptibility. *Trends Biochem Sci* **20**: 397–401

Lairmore TC and Norton JA (1997) Advances in molecular genetics. *Am J Surg* **173**: 37–41

Leach FS, Nicolaides NC, Papadopoulos N, Liu B, Jen J, Parsons R, Peltomaki P, Sistonen P, Aaltonen LA and Nystrom Lahti M et al (1993) Mutations of a mutS homolog in hereditary nonpolyposis colorectal cancer. *Cell* **75**: 1215–1225

Leach FS, Polyak K, Burrell M, Johnson KA, Hill D, Dunlop MG, Wyllie AH, Peltomaki P, de la Chapelle A, Hamilton SR, Kinzler KW and Vogelstein B (1996) Expression of the human mismatch repair *hMSH2* in normal and neoplastic tissues. *Cancer Res* **56**: 235–240

Leung SY, Yuen ST, Chung LP, Chu KM, Chan AS and Ho JC (1996) hMLH1 promoter methylation and lack of hMLH1 expression in sporadic gastric carcinomas with high-frequency microsatellite instability. *Cancer Res* **59**: 159–164

Li M, Zhang ZF, Reuter VE and Cordon Cardo C (1996) Chromosome 3 allelic losses and microsatellite alterations in transitional cell carcinoma of the urinary bladder. *Am J Pathol* **149**: 229–235

Lim PC, Tester D, Cliby W, Ziesmer SC, Roche PC, Hartmann L, Thibodeau SN, Podratz KC and Jenkins RB (1996) Absence of mutations in DNA mismatch

*© 2001 Cancer Research Campaign*

repair genes in sporadic endometrial tumors with microsatellite instability. *Clin Cancer Res* **2**: 1907–1911

Liu B, Parsons RE, Hamilton SR, Petersen GM, Lynch HT, Watson P, Markowitz S, Willson JK, Green J, de la Chapelle A et al (1994) hMSH2 mutations in hereditary nonpolyposis colorectal cancer kindreds. *Cancer Res* **54**: 4590–4594

Lo Muzio L, Nocini P, Mignogna MD, Pannone G, Staibano S, Procaccini M, Mariggio MA, Dolci M and De Rosa G (1999) Immunocytochemical detection of hMSH2 and hMLH1 expression in oral SCC. *Anticancer Res* **19**: 933–940

Loeb LA (1991) Mutator phenotype may be required for multistage carcinogenesis. *Cancer Res* **51**: 3075–3079

Loeb LA (1994) Microsatellite instability: marker of a mutator phenotype in cancer. *Cancer Res* **54**: 5059–5063

Mao L (1996) Genetic alterations as clonal markers for bladder cancer detection in urine. *J Cell Biochem Suppl* **25**: 191–196

Mao L, Lee DJ, Tockman MS, Erozan YS, Askin F and Sidransky D (1994) Microsatellite alterations as clonal markers for the detection of human cancer. *Proc Natl Acad Sci U S A* **91**: 9871–9875

Mao L, Schoenberg MP, Scicchitano M, Erozan YS, Merio A, Schwab D and Sidransky D (1996) Molecular detection of primary bladder cancer by microsatellite analysis. *Science* **271**: 659–662

Modrich P (1991) Mechanisms and biological effects of mismatch repair. *Annu Rev Genet* **25**: 229–253

Mourah S, Cussenot O, Vimont V, Desgrandchamps F, Telllac P, Cochant Priollet B, Le Duc A, Fiet J and Soliman H (1998) Assessment of microsatellite instability in urine in the detection of transitional-cell carcinoma of the bladder. *Int J Cancer* **79**: 629–633

Nicolaides NC, Papadopoulos N, Liu B, Wei YF, Carter KC, Ruben SM, Rosen CA, Haseltine WA, Fleischmann RD, Fraser CM, Adams M, Venter J, Dunlop M, Hamilton S, Petersen G, de la Chapelle A, Vogelstein B and Kinzler K (1994) Mutations of two PMS homologues in hereditary nonpolyposis colon cancer. *Nature* **371**: 75–80

Orlow I, Lianes P, Lacombe L, Dalbagni G, Reuter VE and Cordon Cardo C (1994) Chromosome 9 allelic losses and microsatellite alterations in human bladder tumors. *Cancer Res* **54**: 2848–2851

Papadopoulos N, Nicolaides NC, Wei YF, Ruben SM, Carter KC, Rosen CA, Haseltine WA, Fleischmann, RD, Fraser CM, Adams MD, Ventor J, Hamilton S, Petersen G, Watson P, Lynch H, Peltomäki P, Mecklin J-P, de la Chapelle A, Kinzler K and Vogelstein B (1994) Mutation of a mutL homolog in hereditary colon cancer. *Science* **263**: 1625–1629

Parkin DM, Pisani P and Ferlay J (1993) Estimates of the worldwide incidence of eighteen major cancers in 1985. *Int J Cancer* **54**: 594–606

Parsons R, Li GM, Longley MJ, Fang WH, Papadopoulos N, Jen J, de la Chapelle A, Kinzler KW, Vogelstein B and Modrich P (1993) Hypermutability and mismatch repair deficiency in RER+ tumor cells. *Cell* **75**: 1227–1236

Parsons R, Li GM, Longley M, Modrich P, Liu B, Berk T, Hamilton SR, Kinzler KW and Vogelstein B (1995) Mismatch repair deficiency in phenotypically normal human cells. *Science* **268**: 738–740.

Peltomaki P (1997) DNA mismatch repair gene mutations in human cancer. *Environ Health Perspect* **105**: 775–780

Peltomaki P and de la Chapelle A (1997) Mutations predisposing to hereditary nonpolyposis colorectal cancer. *Adv Cancer Res* **71**: 93–119

Risinger JI, Berchuck A, Kohler MF, Watson P, Lynch HT and Boyd J (1993) Genetic instability of microsatellites in endometrial carcinoma. *Cancer Res* **53**: 5100–5103

Rosin MP, Cairns P, Epstein JI, Schoenberg MP and Sidransky D (1995) Partial allelotype of carcinoma in situ of the human bladder. *Cancer Res* **55**: 5213–5216

Sambrook J, Fritsch EF and Maniatis T (1989) *Molecular cloning: A laboratory manual*. Cold Spring Harbor Laboratory Press: Cold Spring Harbor, NY

Speicher MR (1995) Microsatellite instability in human cancer. *Oncol Res* **7**: 267–275

Steiner G, Schoenberg MP, Linn JF, Mao L and Sidransky D (1997) Detection of bladder cancer recurrence by microsatellite analysis of urine. *Nat Med* **3**: 621–624

Thibodeau SN, Bren G and Schaid D (1993) Microsatellite instability in cancer of the proximal colon. *Science* **260**: 816–819.

Thibodeau SN, French AJ, Roche PC, Cunningham JM, Tester DJ, Lindor NM, Moslein G, Baker SM, Liskay RM, Burgart LJ, Honchel R and Halling KC (1996) Altered expression of hMSH2 and hMLH1 in tumors with microsatellite instability and genetic alterations in mismatch repair genes. *Cancer Res* **56**: 4836–4840

Tomlinson IP, Ilyas M and Bodmer WF (1996) Allele loss occurs frequently at hMLH1, but rarely at hMSH2, in sporadic colorectal cancers with microsatellite instability. *Br J Cancer* **74**: 1514–1517

Uchida T, Wang C, Wada C, Iwamura M, Egawa S and Koshiba K (1996) Microsatellite instability in transitional cell carcinoma of the urinary tract and its relationship to clinicopathological variables and smoking. *Int J Cancer* **69**: 142–145

Varley JM, McGown G, Thorncroft M, James LA, Margison GP, Forster G, Evans DGR, Harris M, Kelsey AM and Birch JM (1999) Are there low-penetrance *TP53* alleles? Evidence from childhood adrenocortical tumours. Am J Hum Genet **65**: 995–1006

Wada C, Shionoya S, Fujino Y, Tokuhiro H, Akahoshi T, Uchida T and Ohtani H (1994) Genomic instability of microsatellite repeats and its association with the evolution of chronic myelogenous leukemia. *Blood* **83**: 3449–3456

Wieland I, Ammermuller T, Bohm M, Totzeck B and Rajewsky MF (1996) Microsatellite instability and loss of heterozygosity at the hMLH1 locus on chromosome 3p21 occur in a subset of nonsmall cell lung carcinomas. *Oncol Res* **8**: 1–5

 *© 2001 Cancer Research Campaign*
